# Factors with the strongest prognostic value associated with in-hospital mortality rate among patients operated for acute subdural and epidural hematoma

**DOI:** 10.1007/s00068-020-01460-8

**Published:** 2020-08-10

**Authors:** Bartłomiej Kulesza, Marek Mazurek, Adam Nogalski, Radosław Rola

**Affiliations:** 1 Chair and Department of Neurosurgery and Paediatric Neurosurgery, Medical University in Lublin, Independent Public Clinical Hospital No. 4 in Lublin, Jaczewskiego 8, 20-954 Lublin, Poland; 2 Chair and Department of Trauma Surgery and Emergency Medicine, Medical University in Lublin, Independent Public Clinical Hospital No. 1 in Lublin Poland, Stanisława Sztaszica 16, 20-400 Lublin, Poland

**Keywords:** Traumatic brain injury, Epidural hematoma, Subdural hematoma, In-hospital mortality rate

## Abstract

**Introduction:**

Traumatic brain injury (TBI) still remains a serious health problem and is called a “silent epidemic”. Each year in Europe 262 per 100,000 individuals suffer from TBI. The most common consequence of severe head injuries include acute subdural (SDH) and epidural hematomas (EDH), which usually require immediate surgically treatment. The aim of our study is to identify factors which have the strongest prognostic value in relation to in-hospital mortality rate among of patients undergoing surgery for EDH and SDH.

**Patients and methods:**

Cohort included 128 patients with isolated craniocerebral injuries who underwent surgery for EDH (28 patients) and SDH (100 patients) in a single, tertiary care Department of Neurosurgery. The data were collected on admission of patients to the Emergency Department and retrospectively analyzed. The following factors were analyzed: demographic data, physiological parameters, laboratory variables, computed tomography scan characteristics and the time between trauma and surgery. Likewise, we have investigated the in-hospital mortality of patients at the time of discharge.

**Results:**

We found that the factors with the strongest prognostic values were: the initial GCS score, respiratory rate, glycaemia, blood saturation, systolic blood pressure, midline shift and type of hematoma. Additionally, we proved that a drop by one point in the GCS score almost doubles the risk of in-hospital death while the presence of coagulopathy increases the risk of in-hospital death almost six times.

**Conclusion:**

Most of the factors with the strongest prognostic value are factors that the emergency team can treat prior to the hospital admission. Coagulopathy, however that has the strongest influence on in-hospital death rate can only be efficiently treated in a hospital setting.

## Introduction

Traumatic brain injury (TBI) still remains a serious health and socioeconomic problem being called a “silent epidemic” because the effects of trauma are often not immediately visible [1, 2]. Moreover, TBI usually affects young adults and results in high mortality or severe disability. Each year in Europe 262 per 100,000 individuals suffer from TBI [3, 4]. The most common consequences of severe head injuries are extra-axial hemorrhagic lesions such as an acute subdural (SDH) and epidural hematoma (EDH), which usually require immediate surgically treatment [4–6]. ‘No head injury is too severe to despair of, nor too trivial to ignore’—this the aphorism of Hippocrates shows that estimating the prognosis after head injury has been and remains difficult [7]. Most of the factors associated with mortality, however were analyzed on the basis of all types of TBI, not specifically in patients with EDH and SDH. The aim of our study is to identify factors which have the strongest prognostic value in relation to in-hospital mortality rate among of patients who undergo surgery for acute extra-axial hematoma, i.e. EDH and SDH.

## Patients and methods

**Fig. 1:**
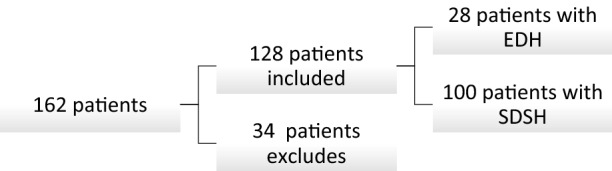
Patients inclusion scheme

The study cohort included 128 patients with isolated craniocerebral injuries. The patients were divided into two groups, namely a group of 28 patients operated on due to epidural hematoma (EDH group) and a group of 100 operated on due to acute subdural hematoma (SDH group). All of the patients were operated and treated in the Department of Neurosurgery and Pediatric Neurosurgery of the Independent Public Clinical Hospital No. 4 (IPCH 4) in Lublin, from 1.10.2014 to 31.08.2017. All patients underwent craniotomy and hematoma evacuation. During this period, 162 patients underwent surgery for extra-axial hematoma whereas 34 patients were excluded from the study due to: the lack of complete medical documentation, incomplete laboratory tests or the lack of formal description of the computed tomography of the head and patients undergoing decompression craniectomy (Fig. 1).

All of the data were collected retrospectively based on the admission data from the Emergency Department (ED). All of the physiological factors were collected up to 10 min after the patient’s arrival in ED. Blood was taken for laboratory tests and computed tomography of the head was performed up to 30 min after arrival in ED. The following variables were analyzed: demographic data (gender and age), physiological factors, laboratory variables, computed tomography scan characteristics and the time between trauma and surgery. Physiological factors included initial GCS score, pupil reaction to light, saturation, systolic blood pressure (SBP), heart rate (HR) and respiratory rate (RR). Laboratory variables included: the number of white blood cells (WBC), hemoglobin (HGB) value, number of platelets (PLT), glycemia value, sodium concentration, and coagulopathy and alcohol levels. Each patient included in the study had a computed tomography (CT) of the head on admission. The study aimed at particular characteristics from the CT, such as the present skull fracture, subarachnoid hemorrhage (SAH), intraventricular hemorrhage (IVH), cerebral contusion, maximum thickness of the hematoma, midline shift (MLS) and state of basal cisterns. The midline shift and maximum thickness of the hematoma were calculated using the OsiriX version 2.5 program based on cross sections from the pre-operative CT scan. The last factor was the time between the injury and the surgery. The in-hospitality mortality of patients was assessed at the time of discharge. Statistical analysis correlated all of the aforementioned factors with in-hospital mortality rate.

### Statistical analysis

In univariate analysis, the Chi^2^ homogeneity test was performed to detect differences in unrelated qualitative characteristics between groups. In multivariate analysis, the logistic regression analysis was used to assess the factors with predominant prognostic value of the in-hospital mortality. If applicable, for comparison of two independent variables the Mann–Whitney *U* test was implemented. A significance level of *p* < 0.05 was assumed indicating the existence of statistically significant differences. In the logistic regression analysis, all the studied factors were considered. For the obtained model, the Chi^2^ value for the difference between the current model and the model with only the free expression was highly statistically significant (*p* < 0.00001). The database and statistical research were based on the STATISTICA 13.0 computer software (StatSoft, Poland).

## Results

The mean age of patients with EDH was 38.81 ± 13.37 years and it was significantly lower than the mean age of patients in SDH group—57.86 ± 18.26 years (*p* = 0.00001). Men were hospitalized most often than women. The patients with SDH had lower GCS scores than those in EDH group (*p* = 0.004). Death during hospitalization was more frequent in patients with subdural hematoma (45%) when compared to the group with epidural hematoma (10.71%), (*p* = 0.0009) (Table 1). The time to death (in-hospital mortality) was slightly longer in the EDH group (median 4 days) in comparison to the SDH group (median 3 days). The difference was not statistically significant (*p* = 0.71), though (Fig. 1).

**Table 1:** Comparison of factors with* p* < 0.05 in both groups

Factors		EDH (*n* = 28)		SDH (*n* = 100		Statistical analysis
		*N*	%	*n*	%	
Gender						
Female		2	7.14	14	14.00	*p* = 0.00001
Male		26	92.86	86	86.00	
Age						
< 35 years old		10	35.71	14	14.00	
36–60 years old		17	60.71	42	42.00	
> 61 years old		1	3.57	44	44.00	
GCS score						
3–8 score GCS		9	32.14	60	60.00	*p* = 0.004
9–12 score GCS		7	25.00	25	25.00	
13–15 score GCS		12	42.86	15	15.00	
In-hospital mortality		3	10.71	45	45.00	*p* = 0.0009

*p* statistical value

### Evaluation of the factors showing the strongest prognostic value regarding in-hospital mortality rate

The factors that entered the model and had a significant relationship with the mortality rate at the level of *p* < 0.05 were: the initial GCS scale, type of hematoma, maximum thickness of hematoma, status of basal cisterns and coagulopathy. The rest of the tested factors with a level of *p* ≥ 0.05 did not enter the model. The obtained logistic model is presented below:

$$P \left(X\right)\frac{{e}^{4.834+\mathrm{0,551} \mathrm{initial GCS scale}-3.279 \mathrm{type of hematoma}-\mathrm{maximum thickness of hematoma}-1.696 \mathrm{status of basal cisterns}+1.770 \mathrm{coagulopathy}}}{{1+e}^{4.834+0.551 \mathrm{initial GCS scale}-3.279 \mathrm{type of hematoma}-\mathrm{maximum thickness of hematoma}-1.696 \mathrm{status of basal cisterns}+1.770 \mathrm{coagulopathy}}}$$

The model shows that a drop by four points in the GCS score affects the increased risk of in-hospital death almost twice (1.73). The presence of epidural hematoma increases the survival charter by 0.04 compared to patients with subdural hematoma. Similarly, the smaller the maximum thickness of hematomas by 10 mm, the death risk is almost one time lower (0.88). Normal size basal cisterns increase the survival chance by 0.18, while the presence of coagulopathy (INR > 1.2 or PT > 12.7 s) increases the risk of in-hospital death (almost six times) (5.87). The results obtained are presented in the table below (Table 2).

**Table 2:** Logistic model for the assessment of prognostic factors of in-hospital mortality in both groups

	Constant	GCS scale	Type of hematoma	Maximum thickness of hematoma	Status of basal cisterns	Coagulopathy
Rating	4.8363	0.5506	− 3.2790	− 0.1305	− 1.6964	1.7697
Standard error	2.3359	0.1161	1.4588	0.0531	0.6379	0.8487
*t* (122)	2.0704	4.7408	− 2.2478	− 2.4580	− 2.6592	2.0852
*p*	0.0405	0.0000	0.0264	0.0154	0.0089	0.0392
− 95% CL	0.2118	0.3207	− 6.1670	− 0.2355	− 2.9593	0.0895
+ 95% CL	9.4608	0.7806	− 0.3910	− 0.0254	− 0.4334	3.4500
Chi^2^ Walda	4.2867	22.4751	5.0526	6.0420	7.0716	4.3480

Statistical analysis: Chi^2^ = 91.53; *p* < 0.00001

**Fig. 2:**
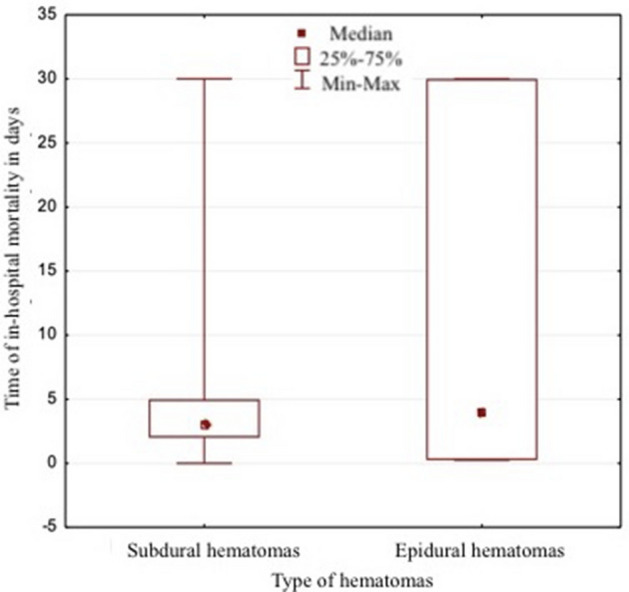
Time in-hospital mortality

As a result of the analysis using Data Mining, the selection and the elimination of variables to assess the prognostic factors of in-hospital mortality, it was shown that the variables included in the table and the figures below are important variables for the assessment of death and survival during hospitalization. The factors with the strongest prognostic value are: the initial GCS score, respiratory rate (below 10 or above 25 breaths per min), hyperglycemia (blood glucose level > 110 mg/dl), saturation (oxygen saturation < 96%), systolic blood pressure (below 90 or above 140 mmHg), midline shift and type of hematoma (Table 3) and (Fig. 2).

**Table 3:** Dominant factors for the dependent variables of the in-hospital mortality

Factors	Chi^2^	*P*
Initial GCS scale	45.05	0.000000
RR	27.88	0.000000
Glycaemia	22.50	0.000002
Saturation	20.19	0.000007
SBP	13.03	0.0003
MLS	25.45	0.0006
Type of hematoma	10.97	0.0009
Pupil reactive	10.80	0.001
IVH	7.65	0.01
SAH	7.07	0.01
Status of basal cisterns	4.65	0.03

## Discussion

### Demographic data

**Fig. 3:**
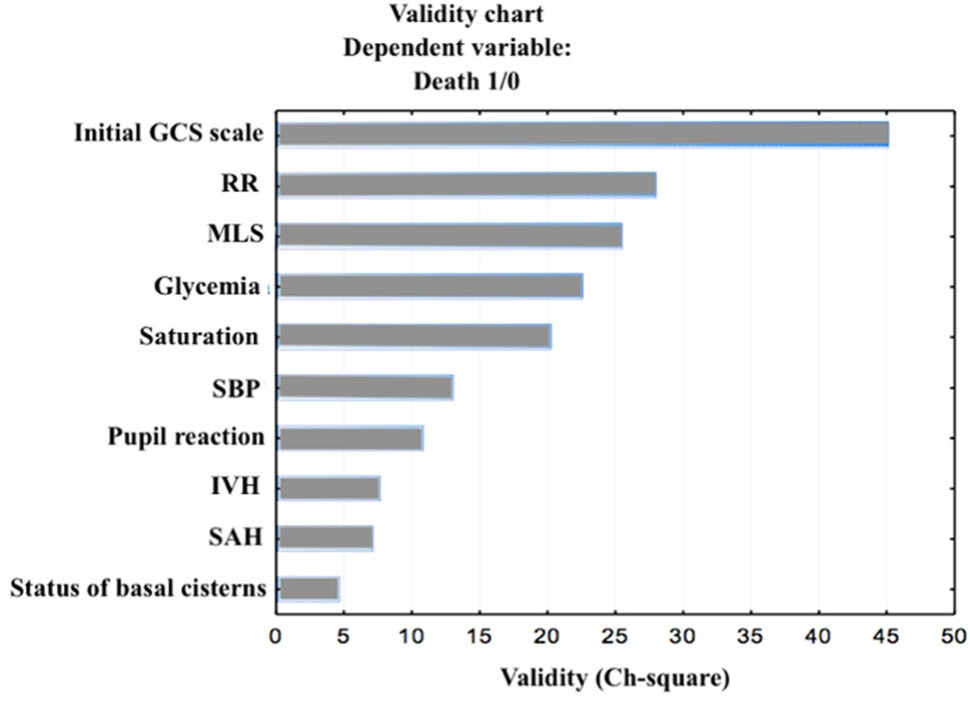
Dominant factors for the dependent variables of the in-hospital mortality

Observations to date have often indicated the impact of demographic factors such as age and gender on the prognosis of patients with TBI. Analysis carried out by Pozzato et al. on 6827 people hospitalized due to TBI showed that the risk of severe trauma is highest at age 15–19 and over 75. The authors estimated that for the latter group it is three times larger than for the general population [8]. A meta-analysis by Hukkelhoven et al. [9] showed that only 15% of patients over 65 years of age have a positive prognosis after severe head injury. Similar conclusions were made by Perel et al. [10] stating that increasing age above 40-years old was associated with higher mortality rate, creating approximately linear function. The relationship between age, unfavorable prognosis and the mortality rate were also seen in the works of other authors [11–17]. It is probably associated with lower regenerative abilities and greater sensitivity of the brain tissue of older people to ischemia as well as frequent coexistence of other diseases in this age group [18–20]. However, our observations did not indicate age among the factors with the strongest predictive value in relation to the in-hospital mortality rate. Some authors pointed to the existence of a difference in the prognosis of traumatic patients depending on the patient’s sex [21]. However, there is strong evidence that gender did not affect the prognosis in TBI [11, 22–26]. This is also confirmed by the results of our observations (Fig. 3).

### Physiological factors

The Glasgow Coma Scale (GCS) is one of the basic tools used to assess the condition of patients after head injuries. It is used not only to assess the victim's state of consciousness, but it can also be useful in predicting the condition of patients in the following days after injury [27]. Likewise, our data prove a strong impact of GCS and the pupil response on the outcome and mortality rate of patients operated on for extra-axial hematoma [28–30]. This relationship was also strongly marked in the observations of other authors [11, 15, 31–34]. Importantly, the multivariate analysis placed GCS score and pupil response to light among the factors with the strongest prognostic value and our studies showed that a drop by one point in the GCS score almost doubles the risk of in-hospital death. However, it should be noted that some authors did not notice similar trends [26].

The Glasgow scale is a very useful tool, but the strategy of dealing with trauma patients cannot depend only on this indicator. It has been proved that the score is related to a number of parameters such as drug and alcohol intoxication, medical sedation, hypotension or hypoxia, often independent of the injury [12]. Hypotension and hypoxia following TBI are recognized as a significant secondary disorder associated with a poor outcome [35]. Petroni et al. found a very strong relationship between low blood pressure and mortality. Hypotension (SBP < 90 mmHg) was associated with 90% mortality rate [36]. The values of SBP higher than 135 or even 150 or lower than 90 mmHg were associated with poorer outcomes [22, 37]. Similarly, our Data Mining analysis placed SBP and saturation amongst the dominant factors that influence mortality with a high statistical significance (*p* < 0.0003).

The impact of other vital signs on the prognosis of patients with TBI was also analyzed. Respiratory rate higher than 25 and lower than 10 increased mortality rates. Similarly, heart rates beyond a normal range are associated with a poor outcome in TBI [37]. The role of respiratory failure was also emphasized in the work of other authors. Osterman et al. [11] showed that mortality among patients after TBI with coexisting respiratory failure reaches 79.3%. It should be emphasized that our research placed RR among the factors closely correlating with mortality (*p* < 0.0000001). Oxygen saturation is yet another indicator directly related to mortality rates following TBI. Kalayci et al. [38] studied the patients undergoing craniectomy for SDH and proved that the saturation less than or equal to 96% was significantly associated with higher mortality rates (*p* = 0.004).

### Laboratory variables

Laboratory variable in TBI patients have also been frequently analyzed in the literature. One of them is stress hyperglycemia, a common finding after the injury [39]. High glucose is a cause of secondary insults for the patients after TBI, and it is associated with a poorer outcome [40]. This trend was also noted in observations carried out by Bobeff et al. The authors showed that patients with glucose levels exceeding 160 mg/dL are at a higher risk of complications after treatment [41]. Similarly, the work of Corbett et al. [42] showed that disorders in this area are associated with higher risk of unfavorable outcome at 18 months after severe TBI. Likewise, in our study hyperglycemia placed among the factors with the strongest prognostic value concerning in-hospital mortality (*p* = 0.000002).

Many authors also considered the relationship between electrolyte balance and the condition of patients after injury. It has been shown that both hypo- and hypernatremia are associated with a poorer outcome, thus sodium revealed a *U* shaped relationship with the outcome [5, 40, 43]. Importantly, hypo-natremia is a relatively infrequent occurrence on admission following TBI. Contrariwise, our study did not confirm the effects of hypo- and hypernatremia on in-hospital mortality.

TBI-associated changes may also be seen in patients’ blood counts. Bobeff et al. [41] showed that abnormalities in hematological parameters such as hemoglobin (Hg), hematocrit (Ht), and red blood cell count (RBC) are an independent risk factor for unfavorable prognosis. Interestingly, the authors also emphasized the role of red blood cell distribution width (RDW) and platelet count disorders in predicting patient mortality. A similar analysis was carried out by Corbett et al. In this study, the association of abnormalities of hemoglobin on admission with a higher risk of adverse prognosis was present during the first 18 months after major TBI. No such relationship was found for the parameters of the white blood cell system (neutrophil and lymphocyte counts), which was previously presented by other authors [42, 44]. Our analysis did not reveal any relationship between abnormalities in blood morphotic systems and in-hospital mortality.

Interestingly, Corbett et al. [42] also showed a relationship between fibrinogen metabolism changes, activated partial thromboplastin time (APTT), international normalized ratio (INR) and disseminated intravascular coagulation score (DIC score) and a greater risk of unsuccessful prognosis within 18 months after the injury. The authors attributed a special predictive value to INR [42]. Likewise, Fuji et al. [45] studying patients who underwent surgery for intracranial hematomas, found that lower values of INR and PTT ensured remarkably better outcomes than higher ones. Coagulopathies, especially changes in prothrombin time and platelet counts, are major determinants of disability and death among the patients with traumatic intracranial hemorrhage [39, 40]. Epstein et al. [46] in their meta-analysis covering 22 studies determined the average percentage of patients with TBI who develop coagulopathy at 35.2%. A convergent correlation was seen in the work of Yuan et al. They estimated that this problem affects 18.6% of patients after isolated TBI, but increases to 30.4% in serious injuries. The authors also showed that patients with severe TBI were characterized by higher INR, prothrombin time (PT), APTT, D-dimer level and lower PLT and fibrinogen levels [31]. Earlier studies defined PT as the indicator most often disturbed in the case of TBI, but APTT seems to correlate better with prognosis and mortality among patients [47–50]. Importantly, our multivariate analysis showed that the presence of any coagulopathy increases the risk of in-hospital death almost six times.

Alcohol consumption was also found to be an important risk factor for TBI, with the prevalence of alcohol intoxication between 20 and 55% at the time of the injury [51, 52]. In our study 31.25% patients were under the influence of alcohol. Alcohol intoxication was associated with a poorer outcome after a severe TBI [15, 52]. On the other hand, it is associated with a decreased mortality [53], thus the relationship between alcohol and the outcome after TBI remains uncertain [53–55]. Our results did not include alcohol in the factors affecting mortality.

### Computer tomography scan characteristics

Imaging studies currently play a key role in the management of head injuries. Strong evidence was found for the midline shift [27, 56, 57] and a greater increase of the midline shift associated with a higher mortality [56, 58]. Ostermann et al. [11] in their observations of 265 elderly patients after TBI showed that midline shifts affect 25% of them. At the same time, they stated that shift over 15 mm is associated with a significantly higher risk of death. In a large analysis of 861 patients after TBI Nelson et al. [59] identified midline shift as the most important risk factor for adverse prognosis. The authors studying patients with EDH and SDH found a higher mortality associated with a greater midline shift, as well as the thickness of hematoma [26, 29, 30]. Our multivariate analysis confirms these relationships between MLS and mortality rate (*p* = 0.0006).

The presence of a traumatic subarachnoid hemorrhage and intraventricular hemorrhage predicts higher mortality [5, 27, 56, 60]. Our multivariate analysis using Data Mining demonstrates that SAH and IVH are both significantly associated with the mortality rate (*p* = 0.01). The next factor associated with in-hospital mortality were the type of hematoma and status of basal cisterns in multivariate analysis (*p* = 0.0009 and *p* = 0.03). In-hospital mortality in EDH group was 10.71% while in SDH group—45%. Grigorakos et al. found the highest mortality rate in SDH (43.75%) in comparison with other post-traumatic changes seen in computed tomography [61]. Khaled et al. found mortality rate among patients with EDH at 10.66% [24]. Status of basal cisterns was closely associated with mortality [10, 22, 58, 62]. Our research showed that all these factors significantly correlated with mortality; in addition the presence of EDH increases the survival chances when compared to patients with SDH. Similarly, the smaller the maximum thickness of hematomas, the death risk is lower. Normal size basal cisterns increase the survival chance by 0.18.

The presence of skull fractures may also be important. On the one hand, thanks to mechanical damage, some of the energy is absorbed, which would otherwise be transferred directly to the sensitive brain tissue. On the other hand, the presence of fractures directly testifies to the large force causing the injury [63]. The skull fracture among TBI patients is associated with an increased risk of neurosurgically-relevant intracranial lesion [64]. Bobeff et al. showed a higher incidence of complications during treatment in patients with linear skull fracture [41]. Our results did not include fractures as factors of unfavorable prognosis.

### Time between injury and operation

Most researchers agree that delaying the moment of surgical treatment implementation negatively affects the survival and prognosis of patients [29, 65, 68, 69]. Matsushima et al. [70] showed that in-hospital mortality was significantly lower in the group of patients operated on within up to 200 min after the arrival at the emergency department (*p* = 0.03). Seelig et al. [71] studying the patients undergoing surgery for SDH, found that the surgery would reduce mortality from 90 to 30% within 4 h. On the other hand, there are still a few studies that did not associate a shorter period of time between the injury and the surgery with the outcome [26, 72, 73]. Our statistical analysis shown no association between time to surgery and in-hospital mortality, but it was reasonable to perform a surgery as soon as possible.

## Conclusion

Amongst the contempororary literature on the patients with a traumatic brain injury, only a few of the studies analyzed a selected group of patients operated on due to the extra-axial hematomas. We were unable to find a study which would collectively analyze all of the factors which we examined in our cohort of patients operated on due to epi and subdural hematomas. Accordingly, we identified a group of the most important factors that highly significantly (*p* ≤ 0.000007) correlate with the in-hospital mortality rate such as: the initial GCS scale, respiratory rate, glycaemia, and saturation. Apart from the GCS scale, these are the factors that can be effectively treated outside the hospital by the ambulance team, which may eventually result in decreased mortality rate. The presence of coagulopathy, however, which increases the risk of in-hospital mortality rate almost six times can be effectively treated only in a hospital setting. Therefore, a prompt transportation to the hospital is also of the paramount importance since the treatment for coagulopathy should be introduced as soon as possible.

Our results require confirmation in other studies on a larger group of patients because this study have significant limitations. First, it included relatively small number of patients operated on for epidural hematoma. Second, no patients conservatively treated due to TBI were included in the study, while a large group of patients after TBI such as those with brain contusions and small intracranial hematomas with no mass effect do not require surgery in the first line treatment. We also did not consider other factors such as blood gas, hematoma volume, C-reactive protein levels, D-dimer and thyroid-stimulating hormone in the study.
